# Transcriptomics and proteomics reveal genetic and biological basis of superior biomass crop Miscanthus

**DOI:** 10.1038/s41598-017-14151-z

**Published:** 2017-10-23

**Authors:** Jiajing Sheng, Xingfei Zheng, Jia Wang, Xiaofei Zeng, Fasong Zhou, Surong Jin, Zhongli Hu, Ying Diao

**Affiliations:** 1 State Key Laboratory of Hybrid Rice, College of Life Sciences, Wuhan University, Luojia Hill, Wuhan, Hubei 430072 P.R. China; 2 School of Pharmaceutical Sciences, Wuhan University, 185 East Lake Road, Wuhan, Hubei 430071 P.R. China; 3 School of Chemistry, Chemical Engineering and Life Sciences, Wuhan University of Technology, 122 Luoshi Road, Wuhan, Hubei 430070 P.R. China

## Abstract

Miscanthus is a rhizomatous C4 grass which is considered as potential high-yielding energy crop with the low-nutrient requirements, high water-use efficiency, and capability of C mitigation. To better understand the genetic basis, an integrative analysis of the transcriptome and proteome was performed to identify important genes and pathways involved in Miscanthus leaves. At the transcript level, 64,663 transcripts in *M*. *lutarioriparius*, 97,043 in *M*. *sacchariflorus*, 97,043 in *M*. *sinensis*, 67,323 in *M*. *floridulus* and 70,021 in *M*. × *giganteus* were detected by an RNA sequencing approach. At the protein level, 1964 peptide-represented proteins were identified and 1933 proteins differed by 1.5-fold or more in their relative abundance, as indicated by iTRAQ (isobaric tags for relative and absolute quantitation) analysis. Phylogenies were constructed from the nearly taxa of Miscanthus. A large number of genes closely related to biomass production were found. And SSR markers and their corresponding primers were derived from Miscanthus transcripts and 90% of them were successfully detected by PCR amplification among Miacanthus species. These similarities and variations on the transcriptional and proteomic level between Miscanthus species will serve as a resource for research in Miscanthus and other lignocellulose crops.

## Introduction

Increased levels of atmospheric carbon dioxide (CO_2_) have been a serious concern among politicians and citizens because of the frequent occurrence of smog and hurricanes. The Kyoto Protocol is a first attempt by the signatory countries to avert these effects. With increasing fossil fuel consumption, biologists and agronomists must develop effective strategies to recycle atmospheric CO_2_. In the natural ecosystem, green plants fix CO_2_ and provide food and energy to non-photosynthetic organisms, including humans. Atmospheric CO_2_ levels had remained relatively stable before worldwide industrialization. Our civilization has prompted people to be dependent on fossil fuel-powered life, and thus living without consuming coal or oil seems impossible. As such, planting fast-growing crops to recycle atmospheric CO_2_ has been the current trend^1^.

In the past 30 years, agronomists in Europe and America have screened a large number of plant species to identify crops with high biomass productivity, high photosynthetic rate, broad adaptability, and minimal requirement for crop management^2–7^. A hybrid derived from a natural cross between *Miscanthus sacchariflorus* and *Miscanthus sinensis*, named *Miscanthus* × *giganteus*, is one of the best biomass crops and has been tested for yield and production efficiency in a large acreage. The highest yield obtained in a field study reached 45 ton/ha^2^. This hybrid is a triploid sterile line and hardly reproductive, with a low propagation rate when the rhizome is used. Furthermore, planting of this crop is labor intensive. Thus, understanding the biological origin and the genetic relationship of *M*. × *giganteus* to related species would enable agronomists to breed ideal biomass crop.

Photosynthesis and cell wall assembly are essential for the growth of *Miscanthus* plants and for obtaining high biomass yield. As such, understanding the mechanisms underlying biosynthesis, transport, and storage of photosynthates, as well as processes involved in cell wall assembly, are useful to improve the energy content of feedstock and efficiently produce ethanol from *Miscanthus* biomass. A number of physiological studies have been performed on photosynthesis and cell wall assembly in *Miscanthus*. For instance, compared with other C4 species, *M*. × *giganteus* grown under chill conditions (≤14 °C) presents increased mRNA levels and/or stability of RNAs related to photosynthetic genes^8,9^. During cold acclimation, phenylalanine ammonia-lyase (PAL) and cinnamyl alcohol dehydrogenase (CAD) activities considerably change in the phenylpropanoid pathway, thereby decreasing lignin content^10^. The coordination of C4 photosynthesis and CO_2_-concentrating mechanism in *M*. × *giganteus* and maize in response to transient changes in light quality were further explored^11^.

*Miscanthus* is a non-model species with only a few nucleotide and protein sequences available in public databases. Biomass production in *Miscanthus* remains partially understood. The lack of dedicated functional genomics resources for this species is a bottleneck in understanding the molecular processes underlying the bioenergy qualities. The greatest challenges are the large physical size of sequencing data (approximately 5 Gb in *M*. *sinensis* and 7.5 Gb in *M*. × *giganteus*) and the large copy numbers of even “low-copy” elements (4 to 6 in *Miscanthus*)^12^. Complementary techniques, such as mRNA-based and/or protein-based methodologies, may be employed to elucidate *Misanthus* genes and their potential functions. Small RNAs were also identified in the genome and transcriptome of *M*. × *giganteus* ^13^. A genetic map of MSI was conducted using RNAseq-based markers and two paralogous C4 pyruvate to identify phosphate dikinase (C4-PPDK) loci^14^. High-throughput exome sequencing coupled with SNP mapping was used to rapidly distinguish cultivars related to *M*. × *giganteus* ^15^. A 2-DE protein map was developed using *M*. *sinensis* leaves to identify heat-responsive candidates^16^. These studies will enhance understanding of complex genomes.

**Figure 1:**
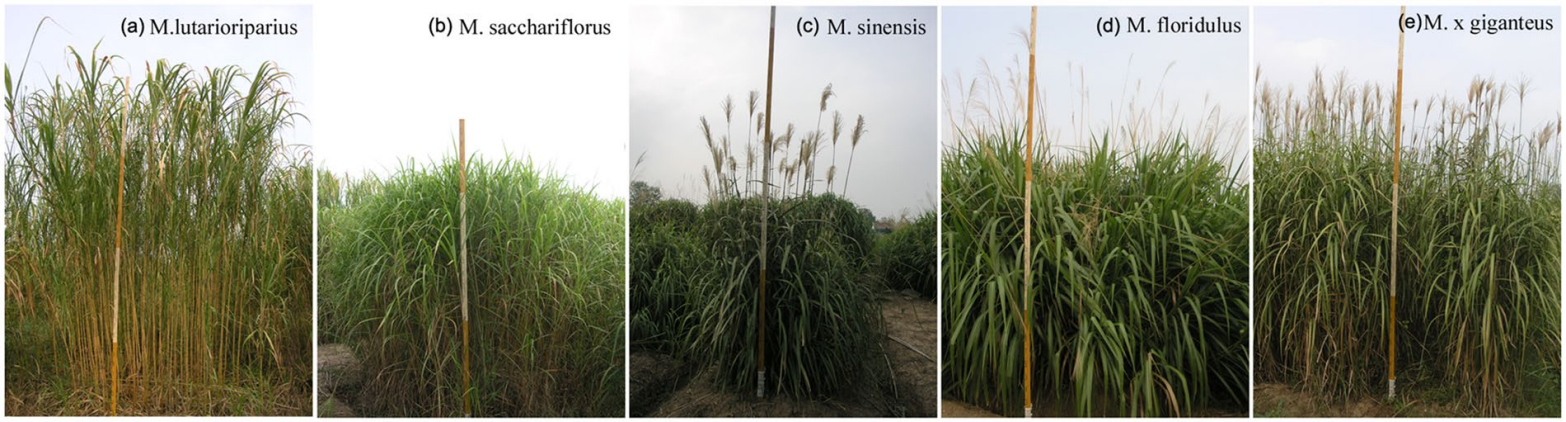
Five Miscanthus species. (**a**) M. lutarioriparius; (**b**) M. sacchariflorus; (**c**) M. sinensis; (**d**) M. floridulus; (**e**) M. × giganteus.

The biological diversity center for *Miscanthus* is located in East Asia. More than 2000 accessions of various species of *Miscanthus* have been collected from all over China and neighboring countries. All *Miscanthus* samples are planted in a germplasm nursery in Hunan Agricultural University and Wuhan University in Central China. Five *Miscanthus* species, including *M*. *sinensis* (MSI), *M*. *floridulus* (MFL), *M*. *sacchariflorus* (MSS), *M*. *lutarioriparius* (MSL), and *M*. × *giganteus* (MGI), are most important biomass breeding resources (Fig. 1). Comparative analysis on photosynthesis and cell wall assembly has been rarely performed at the transcriptome and proteome levels. Leaf is the perfect material used to investigate genetic variation and protein expression associated with biomass accumulation at the early stages of plant development. In this work, comparative deep transcriptome and proteome analyses were performed. The common molecular bases of photosynthesis and cell wall assembly in *Miscanthus* were investigated, and the differences among the species were identified. Phylogenetic relationship was also discussed, and a large amount of SSRs were obtained from *Miscanthus* transcriptome data. Our study provides a valuable resource for accelerating and facilitating functional genomic research in *Miscanthus* and marker-assisted breeding to improve biomass crops.

## Result and Discussion

### Sequencing and de novo transcriptome assembly

**Figure 2:**
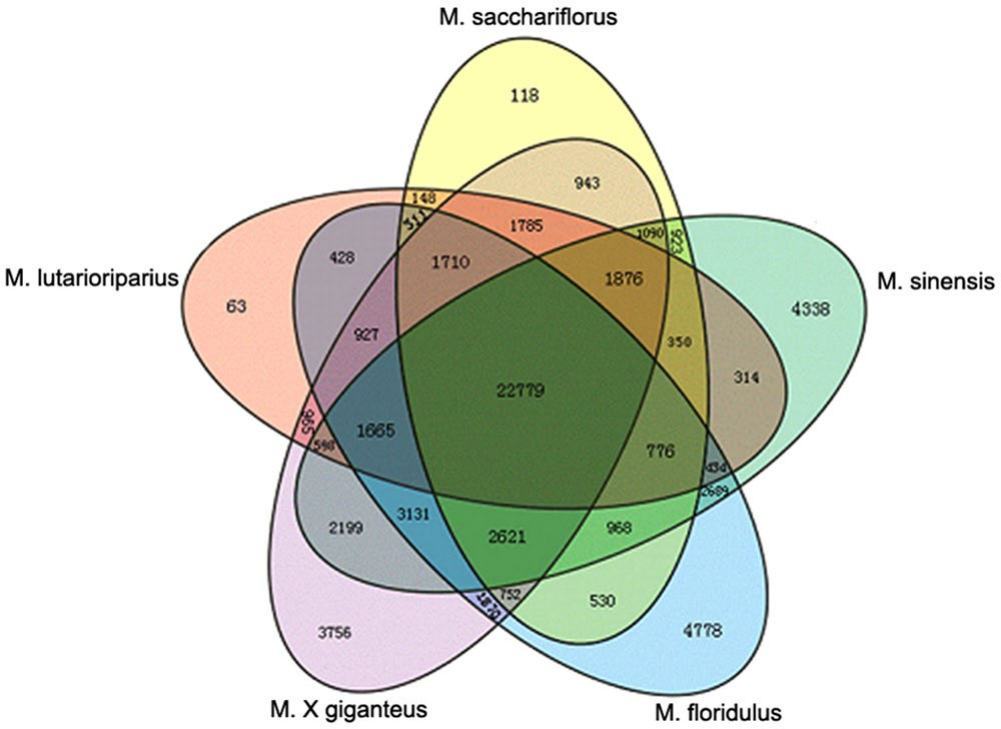
Venn diagram of unigenes identified in five Miscanthus species.

To profile the leaf transcriptome, we isolated mRNA from each of the five *Miscanthus* species, sheared, and subjected to mRNA sequencing. We constructed libraries and analyzed the sequences on the Illumina high-seq. 2000 platform. After cleaning and quality checks, more than 2 Gb sequencing data were obtained from each sample. All sequence read datasets were deposited in the NCBI Sequence Read Archive (SRA) (GenBank accession: SRP051529). A draft *Miscanthus* transcriptome containing a total of 402,164 transcripts were assembled using SOAPdenovo software; these transcripts (known as unigenes) included 64,663 in MSL, 97,043 in MSS, 97,043 in MSI, 67,323 in MFL, and 70,021 in MGI (Table 1). Of the identified total transcripts, 145,257 unique transcripts were generated from the combined transcriptome data of the five samples (defined as M-transcripts) with an average of 471 nt. Approximately 15.69% M-transcripts (22,779 of 145,257) were obtained from all investigated species (defined as MC-transcripts), and the number of species-specific transcripts ranged from 63 in MSL to 4778 in MFL (Fig. 2). MSI, MFL, and MGI contain 4,338, 4,778, and 3,756 unique transcripts, respectively, and MSL and MSS have only 63 and 118 unique transcripts, respectively. Although MSL and MSS shared the most number of common transcripts in this study, these species have been classified by botanists as two different species because of their differences in morphology and distribution^17^.

**Table 1:** Summary of the transcriptome data in Miscanthus leaves.

Samples	Total Nucleotides (nt)	GC percentage	Unigene number	Length of all transcripts (nt)	Mean	The length distribution of transcript				
						100–500 nt	500–1000 nt	1000–1500 nt	1500–2000 nt	>= 2000 nt
M. lutarioriparius	2,424,000,240	54.37%	64,663	27,080,508	419	50,288	11,105	2,221	679	370
M. sacchariflorus	2,405,203,560	54.42%	103,114	36,169,810	351	87,097	12,319	2,530	734	434
M. sinensis	2,311,234,380	54.04%	97,043	32,922,028	339	82,988	11,080	2,069	596	310
M. floridulus	2,358,000,360	54.11%	67,323	27,700,438	411	52,899	11,211	2,222	652	339
M. × giganteus	2,330,000,100	53.43%	70,021	28,714,069	410	55,281	11,464	2,298	624	354
M-transcripts			145257	68,398,166	471	110,154	22,869	6,439	3,008	2,787

### Functional annotation of the transcriptome

To confirm the putative function of the assembled transcripts, we conducted sequence similarity search against four public databases, including NCBI non-redundant (NR), Swiss–Prot protein, Kyoto Encyclopedia of Genes and Genomes (KEGG), and Clusters of Orthologous Groups (COG) by using BLASTx search with a cut-off E value of 10^−5^. Approximately 76% of M-transcripts could be found in the four databases, e.g., NR (48%), Swiss-Prot (27%), KEGG (20%), and COG (13%) (see Supplementary Table S1). Of these M-transcripts, 6,455 transcripts (4.44%) were simultaneously annotated in the four databases. The biological functions of about a quarter of *Miscanthus* transcripts (74,560 of 145,257) remain unknown. The un-annotated 74,560 M-transcripts were considered as the transcripts of the putative unique *Miscanthus* genes. A total of 6,518 transcripts were species-specific and included 875 transcripts in MSL, 1,975 in MSS, 1,456 in MSI, 1,212 in MFL, and 1,000 in MGI.

The biological functions of *Miscanthus* transcripts can be inferred by comparing the sequences with the annotated genomes of well-characterized plant species. Among the identified *Miscanthus* transcripts, about 50% contain orthologous counterparts in the sequenced plant genomes. *Brachypodium* (*Brachypodium distachyon*, 92.84%) and sorghum (*Sorghum bicolor*, 93.18%) showed a large number of matched M-transcripts, whereas *Arabidopsis thanliana*, maize (*Zea mays*), and rice (*Oryza sativa*) presented 80.72%, 78.76%, and 75.71%, respectively (Table 2). Compared with the genomes of these plants, *Miscanthus* genomes appear to have many novel genes.

**Table 2:** Summary of Miscanthus transcripts matched to other plants.

	Arabidopsis thanliana	Brachypodium distachyon	Oryza sativa	Sorghum bicolor	Zea mays
Annotated genes	35386	30129	40353	29448	63540
Matched genes	28564	27972	30550	27440	49800
Percentage	80.72%	92.84%	75.71%	93.18%	78.76%

### GO and KEGG enrichment analysis

**Figure 3:**
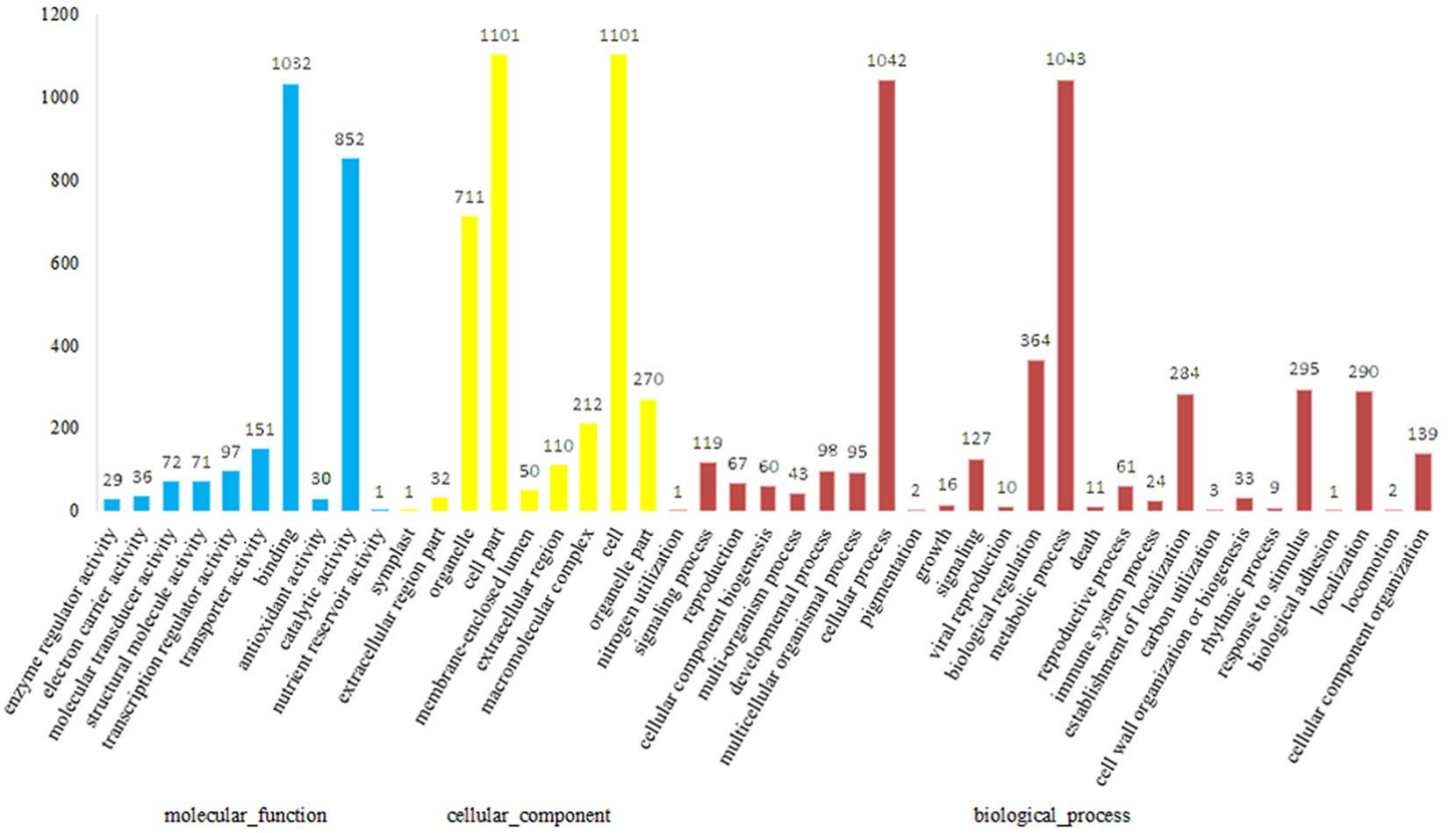
Gene Ontology classification of novel unigenes. Compared with the genomes of Brachypodium distachyon, Sorghum bicolor, Arabidopsis thanliana, Zea mays and Oryza sativa, Miscanthus genomes appear to have many novel unigenes. The results are summarized in three main categories: Biological process, Cellular component and Molecular function. Among them, 1,531 unigenes with BLAST matches to known proteins were assigned to gene ontology.

To gain insights into the functional categorization and metabolic pathways involved in Miscanthus development, total M-transcripts in five species were subjected to enrichment analysis based on GO and KEGG pathways. A total of 1,531 M-transcripts were matched with 3,330 GO descriptions (Fig. 3). In biological processes, 33 M-transcripts are related to cell wall organization or biogenesis, 3 are related to carbon utilization, and 1 is related to nitrogen utilization. In molecular functions, 30 M-transcripts are involved in antioxidant activity and 1 in nutrient reservoir activity. The total M-transcripts were further mapped to the KEGG database and their enrichment of metabolic Pathways were analyzed. Among the top enriched pathways of these pathways, a large number of genes were involved in the pathways related to Metabolic pathways (23.05%), Biosynthesis of secondary metabolites (11.32%), and Plant-pathogen interaction (11.02%) (see Supplementary Table S2). These representations of terms associated with biomass synthesis and metabolic, stress tolerance, and nutrient uptake and utilization may reflect the high adaptability, resistance, and yield of *Miscanthus*.

### Gene validation

According the data from deep sequencing, ten randomly selected unigenes were detected between MSI and MSL. As the results of qRT-PCR shown in Supplementary Figure S1, four unigenes expressed in a similar level between the two species and five differentially expressed. For unigene34227_All, transcripts were only detected in MSL and not found in MSI, which exhibited almost identical expression patterns with sequencing data. The expression patterns of all detected genes show the good agreement between RT-PCR and the deep sequencing method. These results suggest that deep sequencing is an accurate and efficient technique to discover transcripts of genes from *Miscanhtus* species.

### Basic information on the proteomics of *Miscanthus* leaves

**Figure 4:**
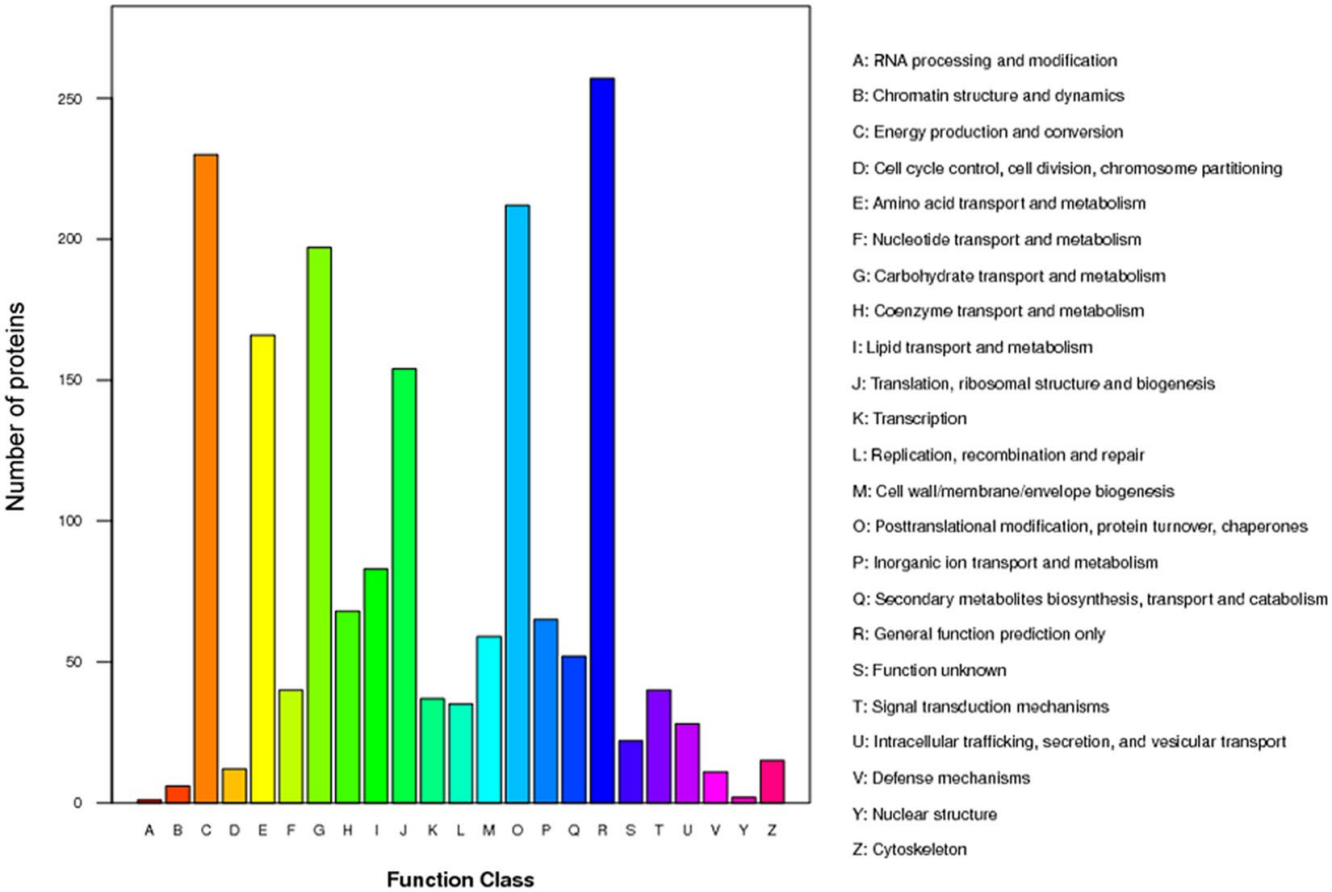
Histogram presentation of clusters of orthologous groups (COG) classification. Among 1,964 Miscanthus proteins, 936 hypothetical or unknown proteins were aligned to COG database to predict and classify possible functions. A total of 643 proeins were assigned to 23 COG classifications.

Although transcriptional profiling has been adopted as the method of choice to study candidate genes putatively involved in plant development processes, biological function is chiefly carried out by proteins, and determination of protein information is mandatory to fully understand the function of a system. To investigate whether *Miscanthus*-specific transcripts were translated into novel proteins, proteome analysis were performed among the five *Miscanthus* species. About 69% of the identified proteins (1335 out of 1964) matched with the *Miscanthus* transcripts, whereas most transcripts did not have their corresponding proteins (Table 3). This finding suggested that the proteome identified the correct protein, but their representation was low. Of the 1,964 *Miscanthus* proteins, 1,028 proteins have been proven to exist in other plants, with 866 proteins annotated as hypothetical proteins and 70 as unknown proteins in the NR plant database (see Supplementary Table S3). Among the identified *Miscanthus* proteins, 654 hypothetical and 2 unknown proteins were matched with sorghum, whereas 103 hypothetical and 48 unknown proteins were matched with maize. Among the 936 hypothetical or unknown proteins, 643 proteins were assigned to 23 COG classifications, which are mostly involved in five basic biological pathways, including translation, ribosomal structure and biogenesis, energy production and conversion, carbohydrate transport and metabolism, amino acid transport and metabolism, and lipid transport and metabolism (Fig. 4).

**Table 3:** Summary of quantified proteins.

Sample	MSS/MSL	MSI/MSL	MFL/MSL	MGI/MSL	MSI/MSS	MFL/MSS	MGI/MSS	MFL/MSI	MGI/MSI	MGI/MFL
Quantified protein	1933	1933	1933	1933	1933	1933	1933	1933	1933	1933
Differentially expressed proteins^*^	83	86	67	82	54	63	86	71	69	61

^*^Fold change ratios > 1.5 and p-value < 0.05. M. lutarioriparius (MSL), M. sacchariflorus (MSS), M. sinensis (MSI), M. floridulus (MFL) and M. × giganteus (MGI).

About 1,964 proteins were mapped to GO terms (see Supplementary Figure S3). The most abundant GO Slims were “cell, cell part, and organelle” for the cellular component, “metabolic process” for the biological process, and “catalytic activity” for the molecular function; these slims were also found in high amounts in the *Miscanthus* transcriptome.

### Phylogenetic analysis of *Miscanthus*

**Figure 5:**
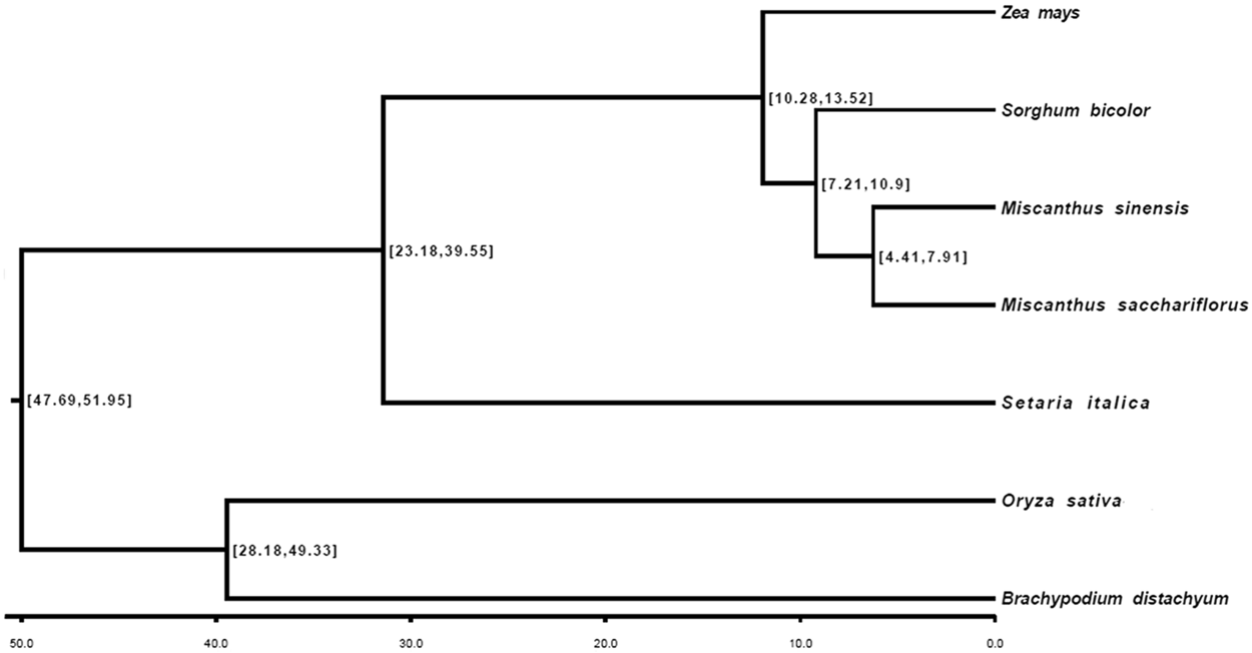
Phylogenetic tree derived from single-copy genes. The numbers refer to the predicted divergence times measured as Myr ago by ML method.

To understand the evolutionary relationship between *Miscanthus* and related grass species, we identified single-copy gene-derived transcripts and used to construct a phylogenetic tree of *Miscanthus* and sequenced monocot species (Fig. 5, see Supplementary Table S4). All the analyzed grasses were clustered in two clades: the first clade includes C4 grasses, such as *Miscanthus*, sorghum, maize, and millets (*Setaria italica*); and the second clade includes C3 grasses, such as rice and brachypodium. The two clades were estimated to have split approximately 54 million years ago. This finding is consistent with previous conclusions inferred from the comparison between a large numbers of orthologous genes^18^. *Miscanthus* is shown to be closely related to sorghum, and their ancestors split from each other approximately 11 million years ago. In the genus *Miscanthus*, divergence between MSI and MSS occurred approximately 7.6 million years ago. Meanwhile, according to the orthologous K-value distribution (see Supplementary Table S5), speciation occurred 5.7 Mya ago for MSL and 6.5 Mya for MSS, MSI, and MFL.

To identify the maternal parent of the natural hybrid MGI, we compared the transcripts of organelle genes (48 from the chloroplast genome and 26 from the mitochondrial genome) between MGI and MSS or MSI (see Supplementary Table S6). Both chloroplast and mitochondrial genes showed that MGI inherited the organelle genome from MSS. Hence, MSS was identified as the maternal parent and MSI as the paternal parent in the natural cross (see Supplementary Figure S4).

### Gene expression within typical C4 biosynthesis pathways of primary carbon fixation in *Miscanthus*

**Figure 6:**
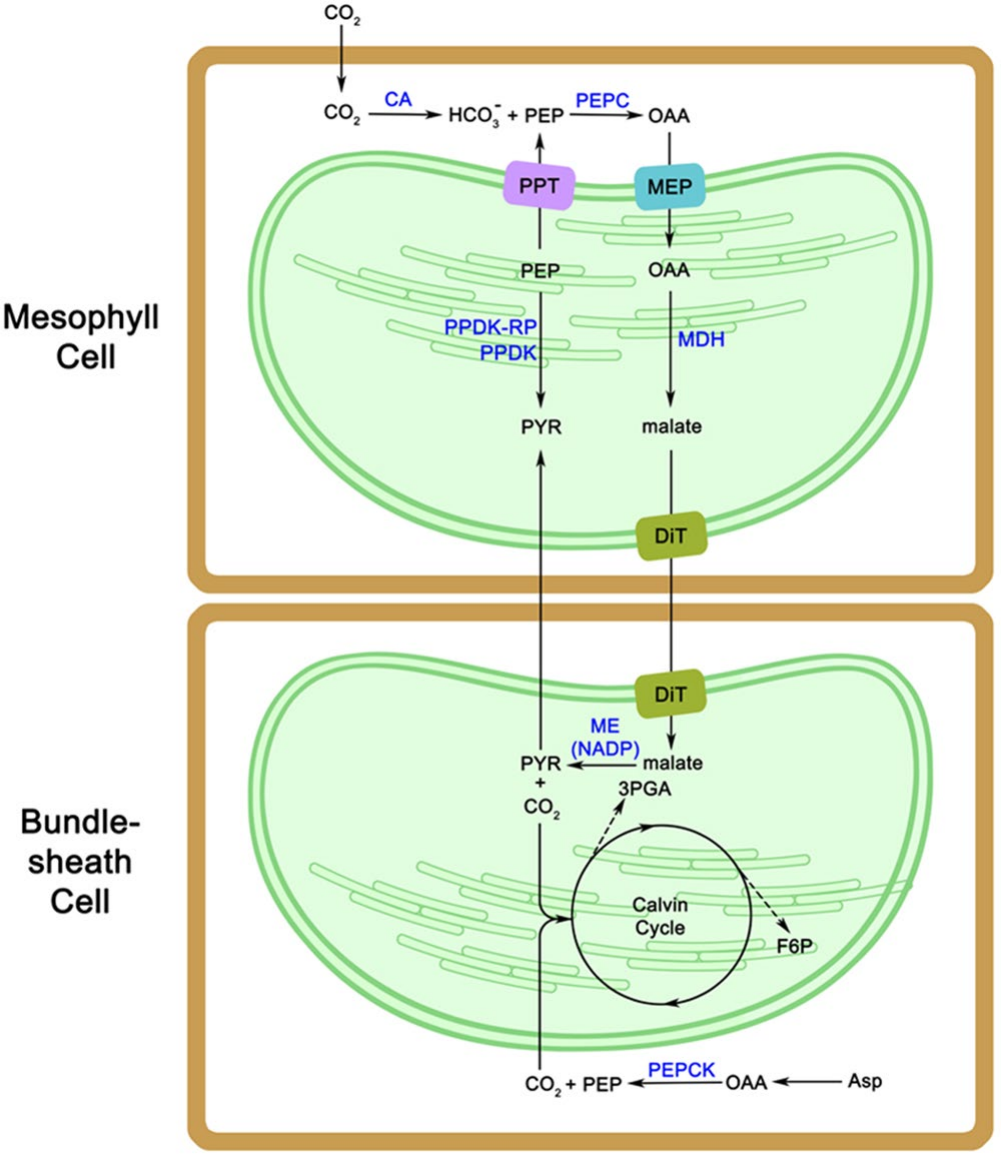
Schema of C4 photosynthetic pathway in Miscanthus. Two cells types, mc and bundle sheath cells, are involved in C4 plant carbon fixation. CA, carbonic anhydrase; PEPC, phosphoenolpyruvate carboxylase; PPT, phosphoenolpyruvate/phosphate translocator; MEP, metabolite transporters at the envelope membrane; MDH, NADP-malate dehydrogenase; PPDK-RP, PPDK regulatory protein; PPDK, pyruvate orthophosphate dikinase; DiT, 2-oxoglutarate/malate translocator; ME (NADP), NADP-dependent malic enzyme; PEPCK, phosphoenolpyruvate carboxykinase; PEP, phosphoenolpyruvate; OAA, oxaloacetate; PYR, pyruvate; 3PGA,3-phosphoglycerate; F6P, fructose-6-phosphate; Asp, aspartate.

*Miscanthus* is a typical C4 species with a two-step carbon cycle that greatly reduces photorespiratory losses. The key photosynthesis genes involved in the C4 metabolic cycle were identified in the investigated *Miscanthus* species based on the known C4 pathways (Fig. 6, see Supplementary Table S7). The genes involved in C4 carbon fixation were all present in the tested *Miscanthus* species as revealed by both transcription and proteome analyses. Carbonic anhydrase (CA) and phosphoenolpyruvate carboxylase (PEPC), which are two important enzymes at the beginning of C4 carbon fixation process, may be directly related to photosynthesis efficiency. In *Miscanthus*, CA transcripts were most abundant in MSI, in which the RPKM value of the total transcripts annotated as CA was fourfold higher than the lowest MGI, whereas the protein abundance of CA was the highest in MGI. Moreover, MGI had the highest abundance of PEPC at the transcript and protein levels. The amount of PEPC proteins in MFL was similar to that in MGI. The enzyme pyruvate orthophosphate dikinase (PPDK) has been implicated in previous researches in the superior cold tolerance of the C4-photosynthesis in M. x giganteus^9^. The transcripts abundance of PPDK was the highest in MSL followed by MGI, while there are no significant different in protein levels between the five species, which may indicate that differential transcripts in the primary metabolism are not translated into differential protein levels. The variation in photosynthesis efficiency among *Miscanthus* species possibly begins during the very early stages of carbon fixation metabolism. In order to verify the actual photosynthetic indexes between five Miscanthus species, their photosynthetic rates and the activities of three C4 enzymes as MDH, PEPC and PPDK in the leaves were measured. The net photosynthetic rate (A) of five Miscanthus species was significantly different (see Supplementary Table S8). The net photosynthetic capacity was highest in MSL (A = 12.47) and MGI (A = 11.18) while other three species (MSS, MSI, MFL) were lower and almost in a similar level (A = 6.2–6.52). On the basis of three C4 enzymes, the relative enzyme activity of the five populations were also variety (see Supplementary Table S9). MGI had the highest PEPC enzyme activity of 26.04 umol mg^−1^ h^−1^, followed by MSL (13.08 umol mg^−1^ h^−1^). The three other species had lower amounts of these enzymes and were ranked in the order of MFL, MSI, and MSS. MSL had the highest PPDK activities, which was approximately equal to MFL. NAD-ME enzyme activity of MGI was highest in five populations, while MSI was the least. The results of these physiological tests were almost consistent with transcription and proteome analyses.

Generally, C4 photosynthesis pathways are defined in three biochemical subtypes based on the subcellular localization and type of C4 acid decarboxylase used by bundle sheath (BS) cells^19^. Although *Miscanthus* and other C4 crop species (maize, sorghum, sugarcane, and switchgrass) are classified under the NADP-ME type, PEP-CK activity was also detected in maize. The transcripts and proteins of all C4 acid decarboxylases, namely, NADP-ME, NAD-ME, and PEP-CK, were found in *Miscanthus*. However, among the three PEP-CK gene transcripts, only one was found in MSI and expressed at low levels, whereas the other two transcripts were expressed at comparatively high levels in all other species. On the contrary, the protein abundance of PEP-CK was the highest in MSI among all *Miscanthus* species. In sugarcane leaves, PEP-CK is one of the most highly expressed bundle sheath transcripts, with relative expression levels higher than that of NADP-malic genes^20^. NAD-ME genes showed fewer transcripts among the three C4 acid decarboxylases in *Miscanthus*, but each transcript had a relative high expression level and the NAD-ME protein was detected in proteome data. Some NADP-ME type C4 plants exhibit a mixed decarboxylation pathway. In *Miscanthus*, the presence of the three C4 acid decarboxylase pathways possibly enhanced CO_2_ transfer from mesophyll cells to bundle sheath cells. We noticed the variation in C4 acid decarboxylase expression between the two sections of the subgenus *Miscanthus*. PEP-CK and NADP-ME were expressed at high levels among the species of Section *Miscanthus* than those of Section *Triarrhena*. This result indicated the possible variation in photosynthesis metabolism between the two sections of *Miscanthus*.

### Cell wall biosynthesis/modification in *Miscanthus*

Cell wall plays an essential role in determining cell size and shape, and thus positively contributes to biomass accumulation. Plant biomass primarily consists of cellulose, hemicellulose, and lignin. Among the five *Miscanthus* species, leaf cellulose content ranged from 39.07% (MGI) to 40.68% (MFL), hemicellulose ranged from 27.04% (MSS) to 37.59% (MSI), and lignin content ranged from 8.35% (MGI) to 11.34% (MFL) (see Supplementary Table S10). Significant differences in hemicellulose and lignin contents, but not in cellulose content, were observed among the five species. A total of 355 genes associated directly or indirectly with cell wall biosynthesis and assembly genes (see Supplementary Table S10; Table S11) were identified through the database of Cell Wall Navigator (CWN; http://bioinfo.ucr.edu/projects/Cellwall/index.pl). These results elucidated cell wall biosynthesis in *Miscanthus*.

Cellulose is a polymer of β-(1,4)-linked d-glucopyranose molecules and serves as the predominant structural polymer in primary and secondary cell walls. Cellulose is synthesized by cellulose synthase superfamily enzymes encoded by CesA genes. We identified transcripts in *Miscanthus* that are homologous to cellulose synthase superfamily in *Arabidopsis*, including AtCesA1 to 10 (see Supplementary Table S11). In the primary wall of *Arabidopsis*, the subunits of cellulose synthase complex are CesA1, CesA3, and CesA6^21–23^, whereas CesA4, CesA7, and CesA8 were found in the secondary wall^24–26^. These six genes, together with CesA2, were all expressed in the five *Miscanthus* species, although CesA5 was found in MSI only and CesA10 was found in MSI, MF, and MGI. The two differentially expressed CesA genes in *Miscanthus* may exhibit species-specific expression.

Hemicelluloses, a heterogeneous group of polysaccharides, strengthen the cell wall via interaction with cellulose and lignin^27^. The structure and abundance of these enzymes vary widely in different species and cell types. Most dicots and noncommelinoid monocots have glucan- or mannan-based polymers as their main cross-linking hemicellulosic polysaccharide. However, in grasses, glucuronoarabinoxylan (GAX) containing a xylan-based backbone with α-l-arabinose (Ara) and α-d-glucuronic acid (α-d-GlcA) substitutions is the predominant hemicellulosic polysaccharide in the cell wall. Enzymes responsible for elongation of the xylan backbone are the GT43 and GT47 families (xyloglucan galactosyltransferases, MUR3). In *Miscanthus*, we identified 17 genes encoding MUR3. Most of these genes (13 in 17) were expressed at a relatively high level in all five species. Thus, we deduced that MUR3 plays a main role in the synthesis of xylan backbone. The members of the cellulose synthase-like (CSL) protein families have been shown to be the case for the other hemicelluloses. In *Arabidopsis*, at least six Csl gene subfamilies (CslA to CslG) were identified and different Csl genes are expressed in different tissues and/or at different developmental stages^28^. In this study, genes homologous to AtCslA, B, C, D, E, and G gene subfamilies, except CslF, were found in *Miscanthus*. The absence of CslF genes, which are involved in the biosynthesis of cell wall (1/3;1/4)-b-D-glucans, has also been observed in rice and other monocots^29^. Only three Csl genes encoding CslA2, CslD3, and CslE1 were expressed in all investigated *Miscanthus* species and the remaining 18 Csl genes were unique or shared by some of them. This result suggested that hemicellulose synthesis may considerably vary among *Miscanthus* species, resulting in the differences in the quantity and composition of hemicelluloses among the species.

Pectins, which are branched hydrated polymers rich in d-galacturonic acid, are believed to influence cell-to-cell adhesion^30^. The middle lamella formed after cell division mainly contains pectins, and most dicots and noncommelinoid monocots contain a considerable amount of pectin. However, glucuronoarabinoxylans (GAX) is thought to replace pectin, to some extent, by controlling the pore size and charging of the cell wall in grasses, such as maize^31^. Thus, the maize cell wall contains low pectins relative to most dicots. We did not detect the transcripts of homologous genes encoding pectins possibly because of the low expression level of these genes. This result may imply that low amount of pectin is possibly deposited in the cell wall of *Miscanthus*.

Lignin is the third component of cell wall and consists of three monomers, namely, phydroxyphenyl (H), guaiacyl (G), and syringyl (S) monolignols. Lignin concentration and composition considerably influence the processing quality when biomass is used as feedstock for biofuel production. *Miscanthus* lignin contains relatively high levels of H units, in addition to G and S units typically found in dicotyledonous angiosperms and most cereals^32^. Although the transcripts of genes encoding the 12 enzymes in monolignol metabolism were identified in this study (see Supplementary Table S12), four of them were not found in protein profiles because of their low abundance; these enzymes include hydroxycinnamoyl CoA shikimate hydroxycinnamoyl transferase (HCT), *p*-coumaroylshikimate 3′-hydroxylase (C3′H), ferulic acid 5-hydroxylase (F5H), and laccase (LAC). HCT and C3′H are key enzymes that separate S-lignin and G-lignin biosynthesis from H-lignin biosynthesis, whereas F5H is specifically involved in S-lignin biosynthesis. The difference in the expression levels of these enzymes lead to the output differentiation among the three monolignol synthesis pathways, with H monolignols producing the highest content, followed by G monolignols, and S monolignols producing the lowest amount. For final enzymes in monolignol synthesis pathways, peroxidase (PER) genes were expressed at a higher level than LAC genes in *Miscanthus*, which indicated that *Miscanthus* prefers PER in monolignol metabolism.

Cell wall contains structural and non-structural proteins. Compared with maize, *Miscanthus* contains structural proteins, such as hydroxyproline-rich glycoproteins, proline-rich proteins, and arabinogalactan proteins, but not glycine-rich proteins. This finding reflected the different composition of the cell wall between *Miscanthus* and maize. Another cell wall protein is expansin 10 (EXP10), which encodes α-expansins that are responsible for rapid growth^33^. A total of 10 homolog genes of EXP10 were found in *Miscanthus*, but only two genes were expressed in all species. MSS showed the least expressed EXP10 genes (4 genes), MSL and MSI had the most expressed genes (7 genes), and MGI expressed 5 genes. Consistent with these observations on growth among the five species, MSL grew fast in height and MSI developed more tillers. The differences in growth among *Miscanthus* species may be partially influenced by the diversity of EXP10.

Meanwhile, a cascade of transcription factors is involved in the regulation of secondary wall biosynthesis. These transcription factors are divided into four categories based on functions, namely, NAC master switches, MYB master switches, downstream transcription factors, and lignin-specific MYBs^34^. Among the identified transcription factors in *Miscanthus* (see Supplementary Table S13), only five transcription factors, namely, SND1, SND2, MYB4, MYB43, and NST1, possibly participate in secondary cell wall metabolism. The SND1 and NST1 master switches, which belong to NAC domain-containing genes, regulate a hierarchy of downstream transcription factors, leading to the activation of secondary wall biosynthesis^35,36^. The downstream transcription factors, including SND2 and MYB43, are specifically expressed in cells that develop secondary cell wall^34^. MYB4 is self-downregulated but is upregulated by light, wounding, or sucrose and is involved in lignin biosynthesis^37^. In this study, MYB master switches were not found in *Miscanthus*. Obviously, a transcriptional regulatory network controls secondary cell walls biosynthesis in *Miscanthus*, resulting in the coordinated activation of secondary cell wall biosynthesis genes in the synthesis, transport, and assembly of secondary cell wall components. Identification of the transcriptional factors and uncovering of the molecular mechanisms underlying secondary wall formation in wood and fibers may help develop novel strategies to genetically modify the cell walls of *Miscanthus* according to our needs.

### Detection of microsatellites

**Figure 7:**
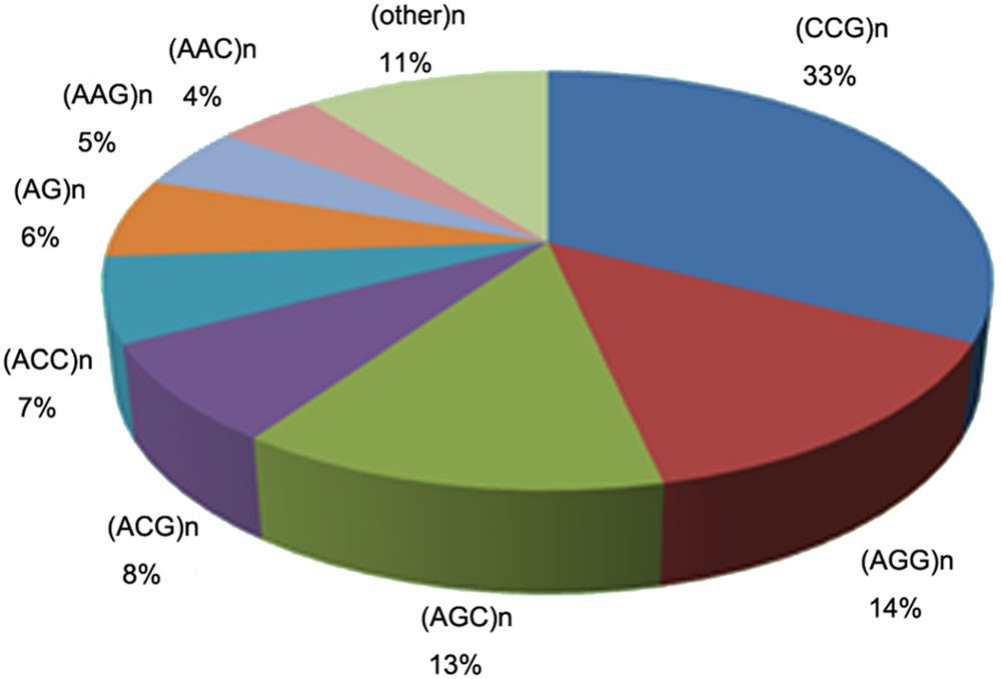
Pattern distribution of the microsatellite repeats in Miscanthus.

Microsatellites or simple sequence repeats (SSRs) are the most preferred types of molecular markers because of their high polymorphism and ubiquitous distribution in genomes^38^. A total of 10,694 potential SSRs were identified using the MISA Perl script (http://pgrc.ipk-gatersleben.de/misa/) in the M-transcripts, ranging from 3,248 (MGI) to 4,499 (MSS) SSRs (Table 4). The tri-nucleotide repeat motifs were the most abundant SSR motif (about 90%) in each *Miscanthus* species (see Supplementary Figure S5). Among the M-transcripts, 406 (7.7%) contained at least ten dinucleotide repeats, whereas 4,617 (87.7%), 167 (3.2%), and 75 (1.4%) contained at least five repeats for tri-, tetra-, and pent nucleotide repeats, respectively. The tri-nucleotide motif, including (CCG)n (33%), (AGG)n (14%), (AGC)n (13%), (ACG)n (8%), (ACC)n (7%), (AAG)n (5%), (AAC)n (4%), and di-nucleotide (AG)n (6%) repeats were the most common in *Miscanthus* (Fig. 7). A total of 7,929 SSR primer pairs were successfully designed according to their good flanking sequences of SSRs (see Supplementary Table S14). About 199 SSRs were derived, and 178 primer pairs were designed from 2,786 M-transcripts, which had BLAST hits to cell wall synthesis-related genes in cell wall navigator (CWN) database (see Supplementary Table S14)^39^. A total of 90 primer pairs were synthesized to validate these primer sets (see Supplementary Table S14); of which, 81 primer pairs (90%) were successfully amplified in *Miscanthus*. The transferability of these SSRs was proven to be good, with 69 SSR microsatellite primer pairs shared by the five *Miscanthus* species and *Narenga porphyrocoma*. SSR analysis revealed the relationships among the five *Miscanthus* species (see Supplementary Figure S6). The results confirmed the taxonomic categories that are, MSI and MFL were closely related and both belong to section *Miscanthus*, whereas MSS and MSL were clustered together under Section *Triarrhena*. Analysis also revealed MGI to be closer to MSS and MSL. These SSRs were directly related to functional genes, which are valuable molecular markers for evolution, genetics, and breeding research in *Miscanthus*.

**Table 4:** Summary of genic-SSRs searching and primers designing results.

Sample	N. unigenes	N. SSRs	Di-nucleotide	Tri-nucleotide	Tetra-nucleotide	Penta-nucleotide	Hexa-nucleotide	Successful primers	Failed primers
M. lutarioriparius	64663	3415	614	1,602	287	101	811	2347	1034
M. sacchariflorus	103114	4499	905	1,984	378	139	1,093	2916	1574
M. sinensis	97043	3957	819	1,731	356	122	929	2576	1349
M. floridulus	67323	3452	641	1,570	267	99	885	2351	1074
M. × giganteus	70021	3248	663	1,529	231	87	738	2153	1058
M-transcripts	145257	10694	2,237	4,617	901	342	2,597	7929	2644

Search parameters: definition (unit size, min_repeats): 2–6 3–5 4–4 5–4 6–3.

### Differentially expressed genes and implications in *Miscanthus* breeding strategy

To elucidate differences among the four *Miscanthus* species, we identified differentially expressed genes at the transcriptome and proteome levels. We obtained a total of 10 crossed comparisons (see Supplementary Table S16). The transcripts and proteins were divided into three categories: group I includes transcripts only found in transcriptome data, group II includes proteins only found in proteome data, and group III includes proteins found in both transcript and protein profiling. Comparison within group I revealed that MFL/MSS had the most differentially expressed transcripts (38,377) whereas MGI/MSL had the least (19,266). In group II, MGI/MSS and MSI/MSL had the most differentially expressed proteins (47), whereas MSI/MSS had the least (24). In group III, among 1,335 genes/proteins, MGI/MSL had the most undifferentiated gene/protein expression (1,098), whereas MSI/MSL had the least undifferentiated genes/protein expression (811). Interestingly, differenced in transcriptional activity and protein abundance between Sections *Miscanthus* and *Triarrhena* were greater than that within the sections. This finding coincides with the genetic diversity of the species.

In hybrid breeding, the concept of heterotic patterns suggests that breeding material is assigned to genetically divergent heterotic pools. Inter-pool hybrids generally show a higher mid-parent heterosis and hybrid performance for yield than the intra-pool hybrids^40^. In some important crops, differentially expressed genes in inbred lines and their hybrids have been investigated to elucidate the molecular basis of heterosis and predict hybrid performance (HP)^41–43^. In this regard, we predicted the HP of *Miscanthus*, which is an undomesticated crop with diverse genetic and phenotypic characteristics. In contrast to other leading crops, the proper inbred line in *Miscanthus* for heterosis research is not available. Among the tested species, MGI is a natural allotriploid hybrid from MSS and MSI and is a high-yield biomass crop. We defined the differentially expressed transcripts between MGI and its parent species into three patterns: above high-parent (AHP), below low-parent (BLP), and mid-parent level (MPL). RPKM comparison in 18,490 C-transcripts revealed that 6,919 transcripts (37.42%) exhibited AHP in MGI, 5,248 (28.38%) were BLP, and 6,323 (34.20%) were MPL. Meanwhile, abundance comparison of 1,913 proteins revealed that 32.83% (628) proteins belong to AHP, 33.09% (633) to BLP, and 34.08% (652) to MPL. We could not disclose exactly that MGI expressed the majority of the transcripts and proteins in an additive or non-additive manner; however, predominant expression in the hybrids compared with that in the parental species were observed based on transcriptome and proteome analyses. This finding is consistent with our observation of the predominant performance of the hybrid in multiple morphological measurements. GO annotation was conducted on differentially expressed transcripts among MGI, MSS, and MSI (see Supplementary Figure S7). A total of 37,511 transcripts were differentially expressed in MSS and MSI. GO analyses revealed that among these transcripts, 2,589 transcripts are involved in molecular function ontology enriched in binding (1239) and catalytic activity (1,176); 3,520 are involved in biological process enriched in cellular (954) and metabolic processes (1,033); and 5,445 are involved in cellular components enriched in the cell (1,867), cell part (1,716), and organelle (1,536). GO terms between MGI and MSS or MSI were similar to those between MSS and MSI. GO ontology of differentially expressed proteins between each two comparisons among these three species was similar to those of differentially expressed transcripts. Although MSI and MSS are not the true parents of MGI, we could infer that heterosis played an important role in the high biomass productivity of MGI. The allotriploid property dosage effect of polyploidy may partially contribute to the high yield of biomass in MGI.

**Figure 8:**
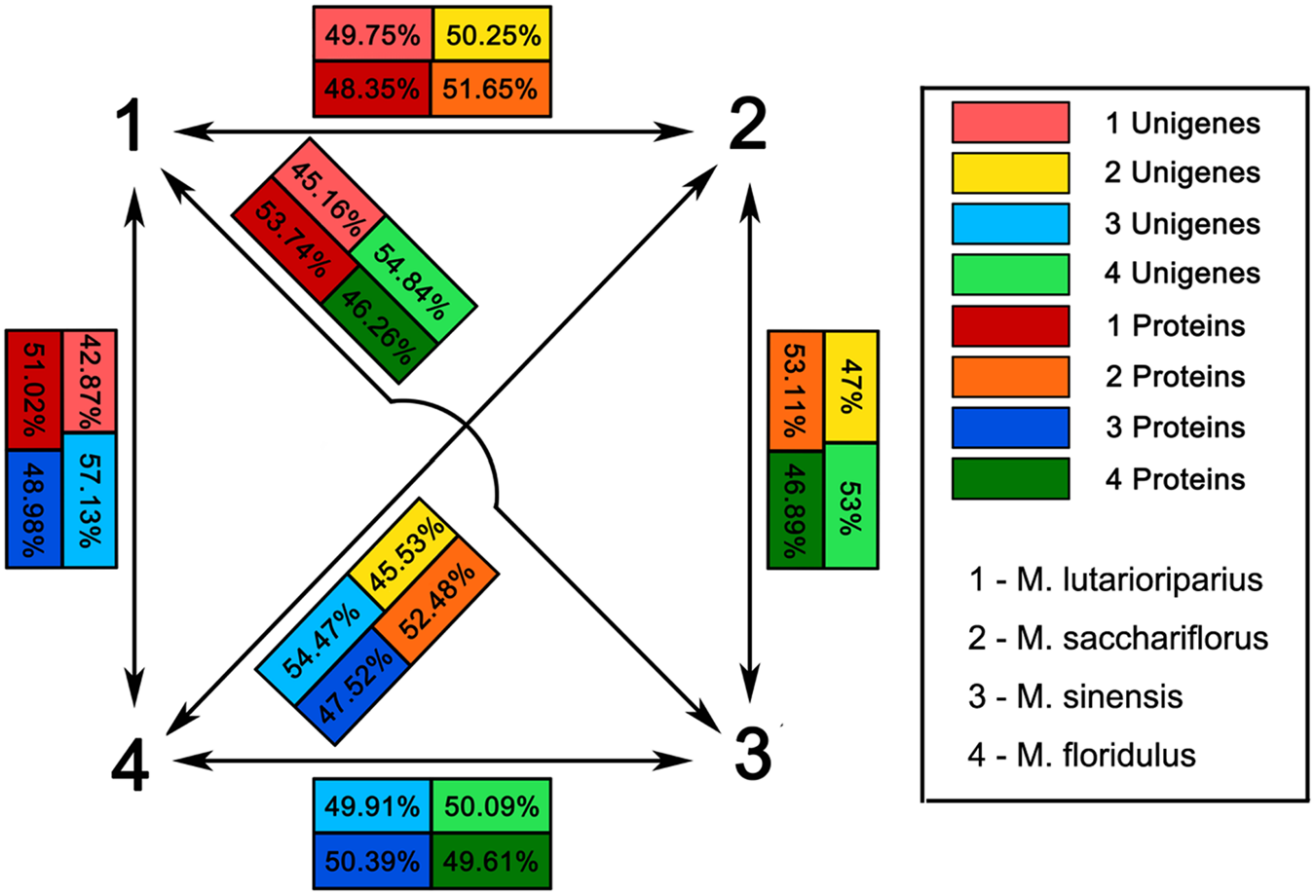
Presentation of transcriptional activity and protein abundance of unigenes that are shared among *M*. *lutarioriparius*, *M*. *sacchariflorus*, *M*. *sinensis* and *M*. *floridulus*. The percentage means the portion of unigenes or proteins that expressed in a high level when comparing within two Miscanthus species.

In this study, four *Miscanthus* species were divided into two groups; the first group comprises MSL and MSS; and the other group consists of MSI and MFL. These two groups are largely different in terms of botanical characters. Obtaining a higher heterosis inter-species hybridization between these two groups is beneficial in *Miscanthus* breeding. MGI is a good heterosis-demonstrating hybrid between MSI (Section *Miscanthus*) and MSS (Section *Triarrhena*). The results (Fig. 8) showed that the pair MSL and MFL or MSI showed larger differences in transcriptional activity and protein abundance than the pairing between MSI and MSS. Hence, the cross between MSL and MFL may produce a hybrid better than MGI. In addition, the combined dosage effect hybridization and polyploidization may be a good approach to develop high-yield biomass varieties of *Miscanthus*. In particular, triploid hybrids exhibit vigorous growth and tolerance to certain stress but cannot produce viable seeds; thus, they pose no risk as weeds^44^.

## Methods

### Plant materials

Representative strains of MSI (MSI-06), MFL (MFL-19), MSS (MSS-02), MSL (MSL-08), and MGI (MGI-01) were selected from our large number of *Miscanthus* germplasm collection. These strains were planted in the greenhouse in Wuhan University. The fifth leaves were harvested from young culms from each representative clone of five selected species. The leaves were flash frozen in liquid nitrogen for RNA and protein extraction.

### RNA sequencing, read assembly, and unigene annotation

Total RNA was extracted from each sample using TriZol reagent (Promega) according to the manufacturer’s instruction. RNA quality was verified using NanoDrop ND-1000 spectrophotometer (Nano-Drop, Wilmington, DE). Five cDNA libraries were synthesized following the recommended Illumina Library Preparation protocol. Each library was labeled with specific tags and sequenced using Illumina HiSeq™ 2000 in Beijing Genome Institute (Shenzhen, China). Raw reads were initially filtered by removing adaptor sequences, duplication sequences, ambiguous reads, and low-quality reads by using a quality cut-off value of 40. Subsequently, de novo assembly of clean reads was performed using a short-read assembling program, namely, SOAPdenovo^45^. The final overlapping transcript set with least “N”s was used for further analysis. Functional annotations for the assembled transcripts were performed by BLAST similarity search against NCBI nr, COG, GO, KEGG (E-value: 10^−5^). Homology searches were carried out by query of the NCBI nonredundant protein database by using BLASTx (E-value, 10^−5^)^43^. Gene names were assigned to each assembled sequence based on the best BLAST hit. The Blast results were then imported into the Blast2GO program to map the sequences into GO terms^46^. WEGO software was used to analyze the GO functional classification for the transcripts^47^. Transcripts were also aligned in the COG database to predict and classify potential functions based on known orthologous sequences^48^. KEGG database was used to analyze metabolic pathways in *Miscanthus* ^49^.

### Analysis of differential gene expression

For quantification of gene expression, we used the “reads per kb per million reads” (RPKM) method, which considers the variations in gene length and total mapped number of sequencing reads and provides normalized values of gene expression. Thus, the expression level of genes between samples could be compared using the RPKM value^50^. “FDR (false discovery rate) ≤0.001 and the absolute value of log2Ratio ≥1” were set as threshold levels to judge the significance of differentially expressed genes (DEGs)^51^.

### Genic-SSR detection, primer design, and maker validation

Genic-SSR detection was performed in a Perl script known as MIcroSAtellite (MISA, http://pgrc.ipk-gatersleben.de/misa). As real mono-nucleotide repeats and single nucleotide error are difficult to distinguish by Illumina sequencing, particularly in the absence of assembled template, genic-SSRs were considered to contain two to six nucleotide motifs with a minimum of 6, 5, 4 and 3 repeats, respectively. Primer 3 software was used to design primers in the flanking regions of SSRs^52,53^. The parameters for designing the PCR primers were set as follows: (1) primer length ranging from 18 bp to 28 bp; (2) PCR product size ranging from 100 bp to 350 bp; and (3) melting temperature between 50 °C and 65 °C with 60 °C as the optimum annealing temperature. According to these parameters, 90 primer pairs (see Supplementary Table S15) were synthesized and used to validate the sets for successful amplification and detect the transferability in genus *Miscanthus*.

### Phylogenetic analysis

Phylogenetic analysis was performed using a set of 13 single-copy genes from six taxa as recommended in the study of Duarte *et al*.^54^. Transcript sequences were translated into amino acid sequences by BioEdit (http://www.mbio.ncsu.edu/BioEdit/bioedit.html) and aligned using MUSCLE ver. 3.6 (Edgar, 2004). Phylogenetic analysis using the maximum likelihood method was performed using PAUP* ver. 4.0b8 (Swofford, 2002)^55^. The orthologs of these genes were identified by the BLAST search of the corresponding *S*. *bicolor* genes at an E-value of 10^−20^ in the transcriptome datasets of *Miscanthus* species. The synonymous (Ks) and non-synonymous substitutions (Kn) rates were estimated using a maximum likelihood method implemented in the CODEML program of the PAML package Version 4.1 (Yang, 2007). The divergence time (T) in millions of years was calculated as T = Ks ÷ (2 × 6.1 × 10^−9^) × 10^−6^ Mya^56^.

### iTRAQ proteome analysis

For proteome analysis, proteins were extracted from the leaves of each sample following the methods used by Breci *et al*.^57^. After protein precipitation, the protein extracts were subsequently reduced, alkylated, and digested with trypsin^58^. Following the manufacturer’s instruction for TRAQ Reagents Multiplex kit (Applied Biosystems, Foster City, CA), peptides of each sample were labeled with iTRAQ tags: 115(MGI), 117(MFL), 118(MSL), 119(MSS), and 121(MSI). The pooled mixtures of iTRAQ-labeled peptides were fractionated through SCX chromatography. The peptides were then subjected to nanoelectrospray ionization, followed by tandem mass spectrometry (MS/MS) in an LTQ Orbitrap Velos (Thermo) coupled online to HPLC. The MS/MS data were analyzed with ProteinPilot Software 4.0 (Applied Biosystems) by using the Paragon algorithm to determine the expressed proteins with relative quantification and p-values. All raw datasets were deposited in the iProX (subproject ID: IPX0001043001). Reference proteins were obtained from the combining database of NR plants and our transcriptome data of *Miscanthus*, which in total contained 1,004,576 proteins. The criteria with fold-change value ≥1.5 and p-value ≤0.05 were used to identify protein with differential expression between any two selected samples^59^.

### Determination of photosynthetic and the activities of three C4 enzymes

In order to understand the difference in photosynthetic indexes between five Miscanthus species, their photosynthetic rates and the activities of three C4 enzymes as MDH, PEPC and PPDK in the leaves were measured. Net photosynthetic rate (A) were measured on three to five intact leaves per species in each treatment. Measurements were made with a portable photosynthetic system CIRAS-3(PP SYSTEMS, US). Actinic light was supplied by lightemitting diodes (38% red light, 37% green lighe, 25% blue light). All measurements were carried out between 09:00 and 10:00 h.

Extraction of enzyme followed the procedure of Genzalez *et al*.^60^. Briefly, approximately 0.2–0.5 g young leaf tissue was ground on ice and 1.5 ml of grinding media consisting of 0.1 M Tris-HCl (pH 7.8), 10 mM MgCl_2_, 1.0 mM EDTA, 20 mm mercaptoethanol and 2% (w/v) PVP-10. Leaf extracts were then filtered through nylon and centrifuged at 10,000 g for 10 min at 4 °C and the supernatant was used for all enzyme assays. PEPC was assayed according to the method of Genzalez *et al*.^60^. Reaction cuvettes contained 50 mm Hepes-KOH (pH 8), 5 mM MgCl_2_, 10mm NaHCO_3_, 0.2 mm NADH, 1.5 units of pyruvate kinase(Sigma). NADP-ME enzyme activities were monitored based on Johnson *et al*.^61^. Assays were carried out in buffer containing 50 mm Tris-HCI (pH 8), 1 mm EDTA, 1 mm MnCl_2_, 1 mM MgCl_2_, 0.33 mm NADP and 5 mm malate. The method of Sugiyama *et al*.^62^ was used to assay PPDK enzyme activities with minor modification. Reaction cuvettes contained 25 mM Hepes-KOH (pH 8.0), 8 mm MgSO_4_, 0.2 mm EDTA, 2 mM pyruvate, 10 mM DTT, 0.2 mm NADH, 2.5 mm (NH_4_)_2_S0_4_, 10 mM NaHCO_3_, 0.5 units of corn PEP carboxylase, 6 units of malate dehydrogenase (Sigma) and 1 mM ATP. Absorbance changes at 340 nm averaged 10/sec and an extinction coefficient of 6.23 × 106 mm^−1^ cm^−1^ was used to calculate enzyme activities in all assays.

### Determination of cell wall composition

The contents of cellulose, hemicellulose and lignin in ten samples of *Miscanthus* species were determined by F-6 cellulose tester (R.Espinar, S.L.) using the Guo *et al*. (2008) method^63^ with minority modifications as follows: ten samples (0.5–1.0 g) of air dried at 65 °C to constant weights and then neutral detergent fibre (NDF), acid detergent fibre (ADF), acid detergent lignin (ADL) and acid-insoluble ash (AIA) content were measured based on the Van Soest (1968) method^64^. The cellulose and hemicellulose content were calculated using the following equation: Cellulose = ADF − ADL − AIA; Hemicellulose = NDF − ADF.

### qRT-PCR Analysis

In order to technically validate the data from deep sequencing, ten unigenes were randomly selected for real-time RT-PCR analysis between MSI and MSL. The specific primers designed with Primer3 software (see Supplementary Table S17)^52,53^. According to the previous studies, ubiquitin gene was used as an internal control^9^. Total RNA was extracted from leaves with RNAprep pure Plant Kit (Tiangen, China). First-strand cDNA was synthesized using RevertAid Reverse Transcriptase (Fermentas) and diluted 20 fold as template. Experiments were carried out using all-in-OneTM qPCR Master Mix (GeneCopoeiaTM, AOPR-1200) with StepOne plusTM Real-Time PCR system (Applied Biosystems). Quantifying the relative expression of the genes in two samples with three replicates were performed using the delta-delta Ct method as described by Livak and Schmittgen^65^.

### Data availability statement

All data generated or analysed during this study are included in this published article (and its Supplementary Information files).

### Accession number

SRP051529IPX0001043001.

## Electronic supplementary material

Supplementary Information
